# Development and characterization of gluten‐free fried dough (*magwinya*) using sorghum flour

**DOI:** 10.1002/fsn3.4362

**Published:** 2024-09-08

**Authors:** Kundai T. Murungweni, Oluwatoyin O. Onipe, Afam I. O. Jideani

**Affiliations:** ^1^ Department of Food Science and Technology, Faculty of Science Engineering and Agriculture University of Venda Thohoyandou South Africa; ^2^ Department of Biotechnology and Food Technology, Faculty of Science University of Johannesburg Doornfontein South Africa; ^3^ Postharvest Handling Group ISEKI‐Food Association Vienna Austria

**Keywords:** fried dough, gluten‐free, guar gum, *magwinya*, physicochemical characteristics, sorghum, xanthan gum

## Abstract

*Magwinya*, a deep‐fried cereal dough, is usually produced using cake or bread wheat flour due to the naturally beneficial proteins found in wheat. In this study, sorghum flour, a gluten‐free cereal grain, was used to produce *magwinya*. The aim was to develop and characterize gluten‐free fried dough using sorghum flour. Xanthan and guar gum hydrocolloids were added to the sorghum flour in ratios of 0.5%, 1.0%, and 1.5% and 1.5%, 2.0%, and 2.5%, respectively, for *magwinya* production. The physicochemical characteristics of the *magwinya* were compared. The hardness of *magwinya* was significantly lower in sorghum: guar gum (SGG) samples due to their high moisture content. Ash and fiber content were also significantly higher in SGG samples. Increasing the level of SGG increased the volume (63.33–66.67 cm^3^). There was no significant difference in the weight of the samples. An increase in diameter (50.33–52.17 mm) was observed in SGG samples. Color analysis showed a significant increase in the L* (Lightness) of the crumb (46.28–49.12) compared to the crust (26.96–30.11) in the SX (sorghum: xanthan gum) and SGG samples. Redness (12.28–13.77) increased with levels, while yellowness (11.86–14.02) decreased with levels in SX and SGG crust. This study's findings are significant as they indicate that guar gum was the better binder for developing cereal fried dough (*magwinya*) from sorghum. These findings could have practical applications in the food industry, particularly in the development of gluten‐free products and in the use of alternative grains like sorghum.

## Graphical abstract

Sorghum fried dough showed interesting findings when compared to wheat fried dough. The hardness of *magwinya* was significantly lower in sorghum fried dough with guar gum (SGG) due to its high moisture content. Ash and fiber content also remained significantly high in SGG samples. Sorghum fried dough had a low‐fat content compared to the wheat counterpart. Considering all the results obtained in this study, sorghum *magwinya* with guar gum showed superiority among the samples.

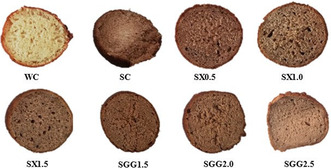

## 1 INTRODUCTION

The production of *magwinya* (a deep‐fried dough) has been done using only cake or bread wheat flour, without the variety use of other different flours like sorghum. Sorghum is a gluten‐free cereal containing many vitamins and minerals, making it an excellent dietary choice for food formulations. *Magwinya*, also known as “fat cake,” is a deep‐fried dough that resembles a doughnut (without a hole), and it is consumed in several sub‐Saharan African and European countries (Mudau et al., 2021). Deep‐fat frying is mostly used in the domestic and industrial processing of food products due to the resulting texture, appearance, and taste of fried products (Abu‐Alruz, 2015; Tabibloghamany et al., 2013). Frying oil can get into the *magwinya* due to the permeable composition of the crust, resulting in an increased fat level of the food. Incorporating the hydrocolloids into the batter formulation, changing the frying processes, and frying medium are efficient strategies to decrease fat uptake in fried foods (Pahade & Sakhale, 2012).

Sorghum (*Sorghum bicolor* (L.) Moench) is a vital cereal grain, especially for communities at risk, providing food to over 500 million people in semi‐arid regions of Africa (ICRISAT, 2009). Sorghum is one of the main sources of energy, vitamins, minerals, and protein for millions of underprivileged people (Garg et al., 2018). Sorghum is a drought‐resistant crop that can withstand harsh conditions during its growth compared to other crops and yields better with good farming (Dykes & Rooney, 2006). Wheat, rye, and barley have gluten—the protein that gives elasticity to dough and helps it rise (Renzetti et al., 2008). Sorghum is a gluten‐free cereal crop that lacks the elasticity and rheology that are necessary when handling the dough for bread and bakery products.

Hydrocolloids offer a variety of functional properties that make them valuable ingredients in the production of baked foods in the food industry. These properties contribute to the stability, texture, and sensory characteristics of baked products (Hedayati et al., 2022; Saha & Bhattacharya, 2010). Xanthan gum exhibits excellent gelling and thickening capabilities, thus providing improved moisture and viscosity retention in doughs and batters (Sworn, 2021). Guar gum acts as a food binder, enhancing the structure and elasticity of dough and preventing stalling (Cappelli et al., 2020). Carrageenan is another commonly used hydrocolloid, and it helps stabilize suspensions and emulsions, contributing to the smooth and uniform texture of creams, fillings, and icings (Imeson, 2012). Pectin is a natural polysaccharide that enables the creation of gels and improves the moisture‐binding ability in fruit‐based jams, jellies, and fillings (Himashree et al., 2022). Cellulose gum is known for its water‐holding capacity, which enhances the overall shelf life of baked foods by delaying stalling and sustaining freshness (Anton and Artfield, 2008; Gladkowska‐Balewicz et al., 2014). By utilizing the functional properties of hydrocolloids, the food industry can form baked products that exhibit desirable stability, texture, and sensory characteristics, eventually meeting consumer expectations.

## 2 MATERIALS AND METHODS

### 2.1 Materials

The materials used to produce sorghum *magwinya* were Monate super mabela sorghum meal (Spar). The nutritional profile of sorghum meal is 354 calories, 7.7 grams of protein, 0.3 g of sugar, 2.9 g of fat, 1000 mg of cholesterol, 7.0 g of fiber, and 15,000 mg of sodium. In addition, sunflower cooking oil, sugar (Selati), salt, yeast (Anchor, Rymco Pty Ltd), xanthan, and guar gum were purchased from a supermarket in Thohoyandou, South Africa.

### 2.2 Sample preparation

Different formulations of sorghum and hydrocolloids (xanthan and guar gum) were used to produce the gluten‐free fried dough (Table 1). The sorghum meal was passed through a 250 μm sieve to obtain fine sorghum flour. Formulations are described as follows: sorghum control (SC), wheat control (WC), sorghum xanthan 0.5 (SX0.5), sorghum xanthan 0.1 (SX1.0), sorghum xanthan 1.5 (SX1.5), sorghum guar gum (SGG1.5), sorghum guar gum 2.0 (SGG2.0), and sorghum guar gum 2.5 (SGG2.5).

**TABLE 1:** Formulations and ingredients quantities (g).

Ingredient	SC	WC	SX0.5	SX1.0	SX1.5	SGG1.5	SGG2.0	SGG2.5
Flour	100	100	100	100	100	100	100	100
Salt	1	1	1	1	1	1	1	1
Sugar	15	15	15	15	15	15	15	15
Yeast	1	1	1	1	1	1	1	1
Xanthan gum	—	—	0.5	1	1.5	—	—	—
Guar gum	—	—	—	—	—	1.5	2.0	2.5
Lukewarm water	75	75	75	75	75	75	75	75

*Note*: Values are the mean of three replications with the standard deviations. Superscripts in the same column with different alphabets significantly differ (*p* < .05). WC and SC are wheat and sorghum control. SX (0.5, 1.0, and 1.5) and SGG (1.5, 2.0, and 2.5) represent xanthan (SX) and guar gum (SGG) variations in the sorghum samples.

### 2.3 Sorghum *magwinya* production process

The ingredients in Table 1 were weighed and mixed manually for 5 min until a homogenous wet, soft, sticky dough formed. The dough was left to ferment at ambient temperature for 45 min. Ensuring uniformity in weight and diameter, the dough was carefully hand‐squeezed into uniform oval shapes and deep‐fried in hot sunflower oil for 5 min at 180°C. After frying, *magwinya* were placed on an absorbent paper to absorb excess oil. They were left to cool down for 30 min at room temperature before analysis (Kwinda et al., 2018).

### 2.4 Experimental design

Two independent factors were investigated, which were the different hydrocolloids that were incorporated in the sorghum flour. The investigation determined the best hydrocolloid suitable for sorghum *magwinya*: xanthan gum and guar gum. The control samples were *magwinya* prepared from 100% sorghum and 100% wheat flour. Six runs were generated to test the level of the two hydrocolloids, which included all two hydrocolloids in combination and three replicates to make a total of six runs. These three levels were used for each hydrocolloid: 0.5%, 1%, and 1.5% for xanthan and 1.5%, 2%, and 2.5% for guar gum. The ratios of sorghum as to hydrocolloid in xanthan gum were (99.5:0.5, 99:1.0, 98.5:1.5) and guar gum (98.5:1.5, 98:2. 97.5:2.5) (Aguilar et al., 2015; Demirkesen et al., 2014). The dependent variables were diameter, weight, volume, hardness, L*, a*, b*, ∆E, moisture, ash, oil, and fiber. All samples were analyzed in independent triplicates.

## 3 METHODS

### 3.1 Weight and volume of sorghum *magwinya* samples

The weight of the samples was measured in triplicates following the method of Onipe et al. (2018). *Magwinya* was cooled to ambient temperature, and weights of three *magwinya* from the same experimental run were collected using a digital weighing balance. The volume of *magwinya* was determined using the rapeseed displacement method according to AACC‐approved Method 10‐05 (AACC, 2000).

### 3.2 Diameter of sorghum *magwinya* samples

The diameter of three *magwinya* balls underwent slicing using a sharp retractable utility knife, and their diameters were subsequently assessed using a Vernier Caliper VC04 manufactured by Walter‐Stern Inc. in NY, USA.

### 3.3 Color determination of sorghum *magwinya* samples

Color analysis was conducted following the methodology outlined by Onipe et al. (2018). The crust and crumb from each sorghum *magwinya* sample were assessed for L* (luminosity), a* (opposition of green and red colors), and b* (opposition of blue and yellow colors). The parameter L* represents the degree of brightness, ranging from black to white, on a scale of 0 to 100. The values for chroma (C), hue angle (H°), and total color difference (**Δ**E) were computed using Equations 1, 2, and 3. The abbreviations Lc (lightness control), ac (hue control along the green to red axis), and bc (hue angle along the blue to red axis) were used.

(1) $$\text{Chroma}=\sqrt{{\left(a*\right)}^{2}+{\left(b*\right)}^{2}}$$

(2) $$\mathrm{Hue}=\tan^{-1}\frac{b}{a}$$

(3) $$\mathrm{\Delta E}=\surd \left[{\left(\text{34𝐿}-\text{34𝐿34𝐿𝑐}\right)}^{2}+{\left(\text{34𝑎}-\text{34𝑎34𝑎𝑐}\right)}^{2}+{\left(\text{34𝑏}-\text{34𝑏34𝑏𝑐}\right)}^{2}\right]$$

### 3.4 Hardness measurement

The sorghum *magwinya* samples were tested for hardness following the method described by Kim et al. (2012) with slight modifications. A TA‐XT plus texture analyzer (Stable Micro Systems Ltd, Godalming, UK) was fitted with a 5 kg load cell. Samples cooled at room temperature for 30 min were subjected to return‐to‐start tests at 40% strain using a 25 mm cylindrical probe (P/26) at 2 mm/s test speed. Hardness was recorded as the peak point in the force deformation curve. Hardness was recorded as the peak positive force (g) in the force deformation curve (Onipe et al., 2018).

### 3.5 Moisture analysis

The moisture level was measured using authorized techniques from AACC (2000) 44–15, 30–25.01, and 08–01.0. After frying, *magwinya* were sliced and subjected to an oven dryer at 105°C until a consistent weight was reached. They were then cooled in a desiccator. The percentage of moisture content was determined by dividing the amount of moisture lost by the weight of the sample using Equation 4.

(4) $$\text{Moisture content}\;\left(\%\right)=\frac{\text{moisture loss}}{\text{sample weight}}\times 100$$

### 3.6 Fat analysis

The fried products were subjected to drying while ensuring no change in weight and then ground using a grinder. Approximately 5 g of *magwinya* sample was placed into cellulose extraction thimbles (25 × 80 × 1.5 mm) from Whatman Intl. Ltd., Maidstone, UK. The extraction process was performed using an automated Soxhlet apparatus, utilizing petroleum ether (40–60°C) for 4 hours, following the AACC method 30–25.01. The fat content was determined using Equation 5.

(5) $$\mathrm{Fat}\;\text{content}\;\left(\%\right)=\frac{\text{weight of extract}}{\text{sample weight}}\times 100$$

### 3.7 Ash analysis

Ash contents were determined in triplicates using approved 08–01.01 of AACC (2000). Silica crucibles were heated, cooled at room temperature, and weighed. Fresh samples (3 g) were weighed into the crucibles, covered, and placed in a muffle furnace at 550°C overnight. Crucibles were cooled in a desiccator and weighed, and the percentage ash was calculated from Equation 6.

(6) $$\mathrm{Ash}\;\text{content}\;\left(\%\right)=\frac{\text{weight of residue}}{\text{sample weight}}\times 100$$

### 3.8 Fiber analysis

Fiber was estimated using method 985.29 of AOAC (2000). Approximately 2 g of defatted *magwinya* samples were treated in a boiling solution of 0.26 N H_2_SO_4_ and 0.23 N KOH. The residue was then separated by filtration, washed, transferred into a crucible, and placed into an oven that was adjusted to 105°C for 18–24 h. The crucible with the sample was weighed and ashed in a furnace at 500°C and weighed again after ashing. The crude fiber was calculated using Equation 7.

(7) $${\text{Crude fiber}}_{=}\;\frac{\text{weight of sample after ashing}-\text{weight of sample before ashing}}{\text{weight of sample}}\times 100$$

### 3.9 Statistical analysis

A one‐way analysis of variance (ANOVA) at 95% confidence level (*p* ≤ .05) using the Duncan multiple range test was used in separating the means of triplicate determinations. The Statistics Packages for Social Sciences (SPSS) version 24 computer software (SPSS 24.0 SPSS Inc., Chicago, 2004) was used for the analysis.

## 4 RESULTS AND DISCUSSION

### 4.1 Weight, diameter, volume and hardness of *magwinya*

The weight change of *magwinya* depends on the oil uptake and moisture loss, whereby a decrease in moisture loss and oil uptake might result in lower weight (Yazdanseta et al., 2015). The weight of *magwinya* ranged from 50.50 to 51.77 g, with no significant difference (*p* > .05) among the samples. Weights of SX and SGG ranged from 50.50 to 51.77 g and 50.60 to 51.27 g. The highest value recorded was 51.77 g, and the lowest was 50.50 g for SX1.5 and SX1.0, respectively. The control samples, WC (51.20 g) and SC (51.03 g), were not significantly different from each other and the other samples. The weight differences might have been attributed to the amounts of hydrocolloid added in SX and SGG samples and the hand‐squeezing technique that can result in the varying weights. The weight of SX was notably higher than that of the other samples. The inconsistencies in the dough texture during frying resulted in irregular shapes (Ndlala et al., 2019).

The mean diameter ranged from 50.00 to 52.17 mm. The recorded results showed that the WC had the highest value (52.17 mm), and the lowest was found in SX0.5 (50.00 mm). This resulted in the gluten found in wheat, which allows the sticky dough to rise well, expand, and form gaseous particles that allow aeration and the dough to rise. Onipe et al. (2019a) stated that when frying the dough, it undergoes expansion due to the pressure gradient created in the inner part of the food. The degree of this expansion depends on the gluten development and the ability of the dough to retain gas during the fermentation. Comparing WC (52.17 mm) and SC (51.00 mm) and the other samples, the significance was because sorghum flour does not have gluten that produces viscoelastic properties in wheat, which therefore makes it difficult to obtain acceptable yeast‐leavened products from 100% sorghum flour (Pontieri & Del Giudice, 2016). The WC (52.17 mm), SGG2.0 (52.00 mm), SC (51.00 mm), SX1.5 (51.67 mm), and SGG2.5 (51.67 mm) were not significantly different (*p* > .05) from each other. The SGG samples had a slightly higher diameter than SX samples. This could be attributed to the fact that guar gum naturally hydrates more slowly and forms larger, more viscous solutions compared to xanthan gum, which hydrates rapidly and forms a more elastic gel (Krstonošić et al., 2021; Wang et al., 2003).

The mean volume values for *magwinya* ranged from 63.33 to 86.67 cm^3^. The volume of SX and SGG samples ranged from a constant value of 66.67 and 63.33 cm^3^ to 66.67 cm^3,^ respectively. Samples containing SX1.5 were not significantly different from each other (*p* > 0.05). The WC (86.67 cm^3^) had a significantly (*p* < .05) higher volume than other samples. The SC (76.67 cm^3^) had a notably higher volume than the SX and SGG samples. Sorghum exhibits lower gelatinization temperature, a thinner bran layer, and amylopectin starch (Dykes & Rooney, 2006). The lack of elasticity (caused by gluten) resulted in a lower capability to allow gas retention and sorghum dough rise during proofing, resulting in reduced volume. A high volume in WC (86.67 cm^3^) was recorded. This was because wheat flour can expand and retain gas due to the presence of gluten compared to sorghum with no gluten. Comparing SX samples to SGG samples, SX had higher constant values that were not significant. The distinction in gas retention characteristics, as seen in Table 2, might be due to a contrast in the permeability and stability of the gas cell wall of the sorghum dough with hydrocolloids as affected by the viscosity of the batter. This implies that adding xanthan gum could affect dough stability during dough proofing (Rosell et al., 2001; Vidaurre‐Ruiz et al., 2019). From Table 2, this might mean that SX has a higher gluten network with better water retention capacity than SGG, resulting in better product volume.

**TABLE 2:** Physical composition of sorghum *magwinya* (fried dough).

Samples	Weight (g)	Diameter (mm)	Volume (cm^3^)	Hardness (g)
WC	51.20^a^ ± 0.36	52.17^a^ ± 1.26	86.67^c^ ± 5.77	2052.13^ab^ ± 74.33
SC	51.03^a^ ± 0.83	51.00^abc^ ± 1.80	76.67^b^ ± 5.77	5328.48^g^ ± 82.54
SX0.5	51.13^a^ ± 0.77	50.00^a^ ± 0.50	66.67^ab^ ± 5.77	4253.91^f^ ± 505.37
SX1.0	50.50^a^ ± 0.56	50.33^ab^ ± 0.76	66.67^ab^ ± 5.77	3680.86^e^ ± 101.14
SX1.5	51.77^a^ ± 0.49	51.67^abc^ ± 0.29	66.67^ab^ ± 5.77	3293.63^d^ ± 157.54
SGG1.5	50.60^a^ ± 0.87	52.00^bc^ ± 0.50	63.33^a^ ± 5.77	1930.74^a^ ± 82.47
SGG2.0	50.80^a^ ± 0.70	51.83^bc^ ± 0.76	66.67^ab^ ± 5.77	2327.10^b^ ± 184. 64
SGG2.5	51.27^a^ ± 0.47	51.67^abc^ ± 0.29	66.67^ab^ ± 5.77	2845.45^c^ ± 121.95

*Note*: Values are the mean of three replications with the standard deviations. Superscripts in the same column with different alphabets significantly differ (*p* < .05). WC and SC are wheat and sorghum control. SX (0.5, 1.0, and 1.5) and SGG (1.5, 2.0, and 2.5) represent xanthan (SX) and guar gum (SGG) variations in the sorghum samples.

### 4.2 Hardness of *magwinya*

**FIGURE 1:**
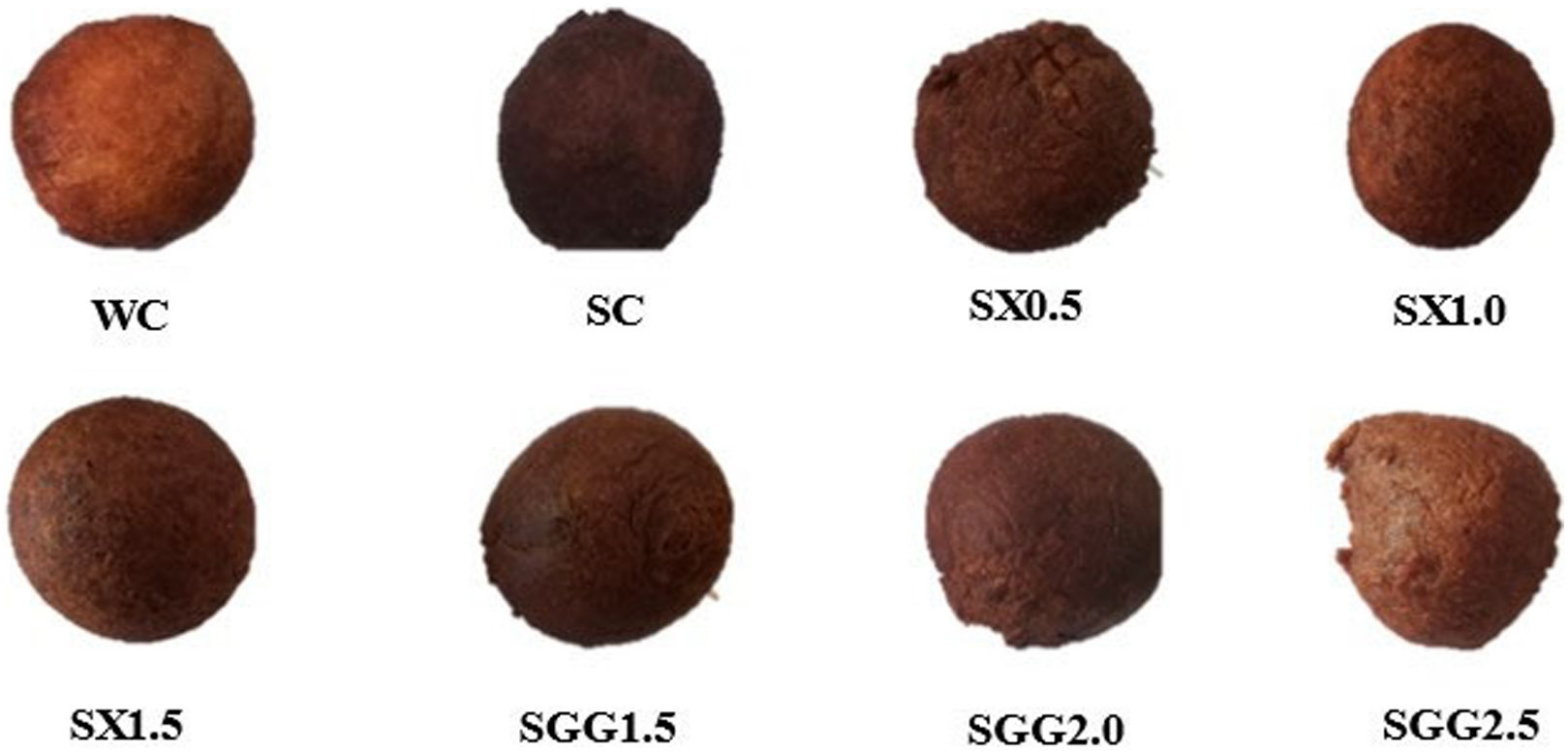
Crust of *magwinya* samples. WC and SC are wheat and sorghum control. SX (0.5, 1.0, and 1.5) and SGG (1.5, 2.0, and 2.5) represent xanthan (SX) and guar gum (SGG) variations in the sorghum samples.

**FIGURE 2:**
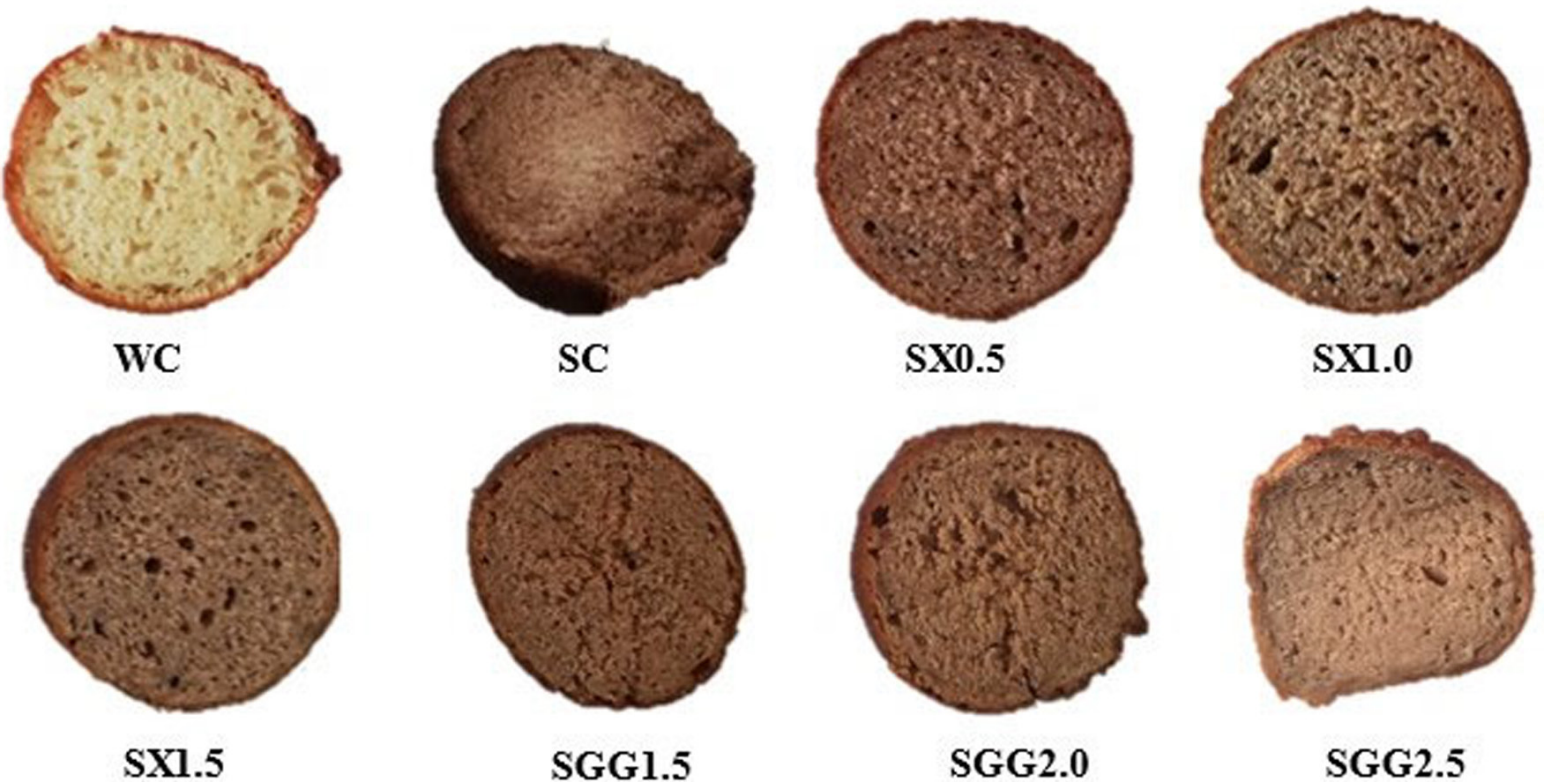
Crumb of the *magwinya* samples. WC and SC are wheat and sorghum control. SX (0.5, 1.0, and 1.5) and SGG (1.5, 2.0, and 2.5) represent xanthan (SX) and guar gum (SGG) variations in the sorghum samples.

One of the most crucial textural criteria is hardness (Kaplan et al., 2020; Mudau et al., 2023). The hardness of a food sample is the ability of a product to resist distortion when an external force is applied to it (Sabanis et al., 2009). Hardness ranged from 1930.73 g to 5328.48 g. Statistically, the SX and SGG samples were different from each other but significantly lower (*p* < .05) than the SC (5328.48 g) sample but higher than the WC (2052.13 g) sample. Baked food hardness during production is affected by factors like moisture content and redistribution and movement of the water in a product (Ndlala et al., 2019). After frying at 180°C for 5 min, the SC (5328.48 g) hardness was the highest due to high moisture loss during frying, which hardened the crust and left the crumb not well cooked, as shown in Figures 1 and 2. Gluten‐starch interaction and starch retrogradation are also important factors contributing to the hardness of fried products (Ndlala et al., 2019). With sorghum being gluten‐free, this might have been a significant contributing factor to the hardness of sorghum samples. Hydrocolloids generally increase viscosity in food staff. Texture changes typically with temperature, concentration, and shear strain rate in a compound relying on the hydrocolloid(s) and materials at hand (Marcotte et al., 2001). SGG *magwinya* samples had lower values compared to SX *magwinya* samples. This could be credited to the fact that guar gum has weaker gel formation, which may result in a softer dough and softer texture in the final product (O'Connor et al., 1981; Tahmouzi et al., 2023). This explains how the increase in viscosity when heat was applied resulted in high hardness values (Table 2). In comparison, Hager et al. (2012) observed that rice flour resulted in a softer and less dense product, which could be due to its lower protein content and starch composition. Therefore, the utilization of rice flour could potentially lead to producing softer *magwinya* as compared to sorghum *magwinya*.

### 4.3 Color properties of *magwinya*

#### 4.3.1 Crust color profile

The caramelized golden‐brown color of the crust and the creamy white crumb are chief factors contributing to how appealing a bakery product can be for a consumer's buying choice and preference (Gallagher et al., 2003). Brown sorghum flour contains several pigments, including flavonoids, carotenoids, and tannins, contributing to its color and nutritional value (Girard & Awika, 2018). Awika (2017) mentioned that the rare pigments found in sorghum have the potential to offer a cost‐effective alternative to natural and stable food coloring. During frying, carotenoids, tannins, and flavonoids can undergo degradation due to the high temperatures, which could lead to the intensity of color change (Grajek & Olejnik, 2010; Nanditha & Prabhasankar, 2008). Due to the dark color of sorghum flour, the crumb and crust color of sorghum *magwinya* was dark brown, the natural color of the sorghum flour used. The color of the crust and crumb of *magwinya* was measured based on the values of Lightness, redness (a*), yellowness (b*), Chroma, Hue, and delta change (∆E). The color properties (L*, a*, and b*) of the crumb of the control were compared to the crumb of the sorghum samples, and the crust of the control was compared to the crust of the sorghum samples.

**FIGURE 3:**
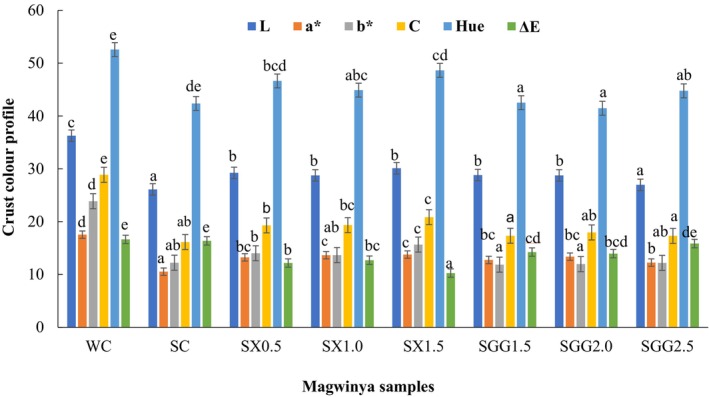
Crust color profile of *magwinya* (fried dough) samples. Values are the mean of three replications. Error bars are standard deviations. Superscripts in the same bar color with different alphabet show a significant difference (*p* < .05). SX (0.5, 1.0 and 1.5) and SGG (1.5, 2.0 and 2.5) represent xanthan (SX) and guar gum (SGG) variations in the sorghum samples.

The lightness of the *magwinya* crust ranged from 26.96 to 36.27. The sorghum dough samples had significantly (*p* < .05) lower L* values as compared to the wheat control (WC) values but markedly higher than the L* values of the sorghum control (SC). Apart from the wheat and sorghum control, the highest L* value was measured for the sample having the highest xanthan gum concentration, and the lowest L* value was for the sample with the highest guar gum concentration. The crust of the sorghum control sample was significantly darker than SX0.5, SX1.0, SX1.5, SGG1.5, SGG2.0 and the WC. This decrease in L* is because of the dark brown color that the sorghum naturally has, which darkens when fried (Yuksel & Kayacier, 2016). Also, chemical reactions such as Maillard and caramelization, which occur during thermal treatment (frying), resulted in the color change in the crust of sorghum *magwinya* (Onipe et al., 2018). The browning level of reactions might have been caused by water activity, temperature, pH, time, and the state of the food system, which keep changing during the frying process (Salvador et al., 2005). Sorghum dough, being naturally dark in color, also influenced the dark color of sorghum samples. SC crust had an unappealing color as it looked burnt and darker after frying for 5 min at 180°C (Figure 3). As frying time increased, the crust got darker.

The results obtained from this study (Figure 3) showed that the redness (a*) values ranged between 10.53 and 17.54; for yellowness values, the b* values ranged from 11.86 to 22.92. The increase in crust yellowness might have been due to the Maillard reactions that took place during the frying of *magwinya* in high temperatures. The study results indicated that the values of the crust a* (redness) and b* (yellowness) of the WC and SC had a significant difference (*p* < .05) from all samples. A substantial increase in a* and b* values of sorghum flour samples was seen, with the highest a* value being 13.77 and b* being 15.66, with the SC showing a significantly lower value of 10.53.

Crust chroma ranged from 16.14 to 28.86, with a significant difference in the SC and WC values. The chroma marks out the intensity of colorfulness appraised by consumers. High chroma values are used to determine the degree of difference of a hue compared to the gray color of a food sample (Pathare et al., 2013). This suggests that WC was more intense than the SC and other samples with lower significant values. There was no significant difference in the SGG chroma samples. The Chroma values of the crumb (14.00–24.05) were lower than the Chroma values of the crust (16.14–28.86). This infers that the intensity of color in the crust was much higher than the crumb due to the sorghum and hydrocolloid incorporation. The *magwinya* crust had significantly higher (*p* < .05) Chroma values when compared to the SC. The crust and crumb chroma were generally lower than the WC but higher than the SC control. This shows that sorghum *magwinya* are less appealing to humans as they have a dark, dull crust color. Moreover, the different porosity of the samples, as seen in Figure 2, could affect chromatic parameters.

Hue angle subjectively describes the conventional color of a product in terms of colors like red or green. As stated by Pathare et al. (2013), hue values that lie between 0° and 90° represent the red hue, whereas values that lie at 90° represent yellow. The crust values of the fried doughs ranged from 41.73° to 52.58°, making them fall under the red hue. The crust and crumb values of hue from this study were below 90°. This suggests that sorghum *magwinya* samples showed redness as the xanthan increased in the crust and crumb but, on the other hand, decreased in the crust and crumb of *magwinya* incorporated with guar gum.

The total color difference (∆E) marks the size of the color change between the test and control samples (Pathare et al., 2013). Color change ranged from crust (10.26–16.36). There was no significant difference (*p* > .05) in the crust of WC and SC color difference, SC and SX crumb samples, and then SGG crumb samples. The color change outcomes showed a clear color difference between *magwinya* wheat and sorghum control and sorghum samples. The scale values of the crust and crumb color profile of the sorghum samples were generally lower, which interpreted that the samples were dark in color. A study done by Onipe et al. (2019b) showed that the consumers preferred *magwinya* control (WC), which had the highest acceptability score on the color appearance in comparison to the fine wheat bran and medium wheat bran *magwinya* used in the study. The color profile of this study assumes that these samples might receive lower ratings of preference from consumers regarding color appearance.

### 4.4 Crumb color profile *magwinya* (fried dough)

L, a*, and b* values for crumb ranged from 45.14 to 67.03, 2.93 to 10.95, and 14.70 to 23.87, respectively. Compared to the WC (L* = 67.03), the sorghum *magwinya* crumb lightness reduced to 45.14 at 2 g of SGG addition. The findings from the study show that, compared to the SC crumb, SX samples increased in lightness, and SGG samples decreased in lightness. This was a result of sorghum having a dark brown color. Processing conditions such as temperature and time used during frying do not affect the color of the crumb. This is because temperature increment does not give Maillard reaction or caramelization process to the crumb. The reduction of crumb lightness is very much related to the effect the source of fiber has on crumb moisture. The higher the moisture, the lower the lightness (Onipe et al., 2018), hence the decrease of L* compared to the WC. There was no significant difference (*p* > .05) between the SC and SX samples and SGG samples.

**FIGURE 4:**
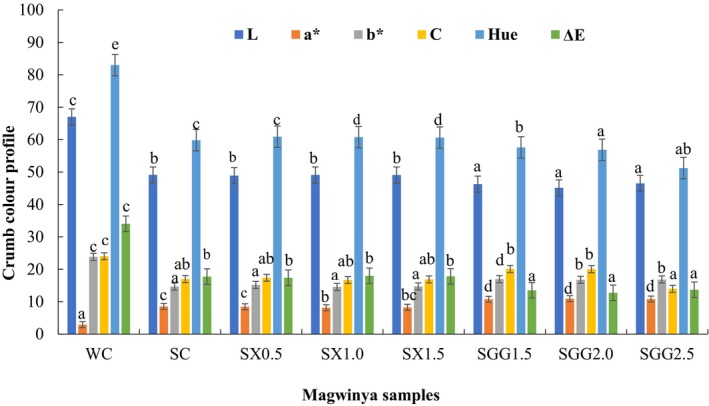
Crumb color profile of *magwinya* (fried dough). Values are the mean of three replications. Error bars are standard deviations. Superscripts in the same bar color with different alphabet show a significant difference (*p* < .05). WC and SC are wheat and sorghum control. SX (0.5, 1.0, and 1.5) and SGG (1.5, 2.0, and 2.5) represent xanthan (SX) and guar gum (SGG) variations in the sorghum samples.

The a* and b*values of the crumb ranged from 2.93 to 10.95 and 14.70 to 23.87 (Figure 4). The SGG samples had no significant difference (*p* > .05). As compared to the WC (2.93) and SC (8.54), the redness (a*) values of SX and SGG samples increased with the level of hydrocolloid. The b* values of SX had no significant difference from SC values. This is also applied to the b* values of SGG. This might have been due to the flour type, the grain structure, and the color of the ingredients, such as the flour. Therefore, the dark brown sorghum used in this study affected the color of the crumb.

### 4.5 Moisture content of *magwinya*

Water evaporation leading to moisture loss is generally a significant phenomenon in fried products (Arshad et al., 2019). The moisture of *magwinya* with SX and SGG ranged from 34.00% to 39.06%, and there was a significant increase in the moisture content with the rise in the level of hydrocolloids incorporated. Yazdanseta et al. (2015) reported high moisture retention when guar gum was added to yeast‐raised doughnuts; in this study, SGG2.5 showed the highest moisture content of 39.06%. This can be explained by its gel formation with sorghum starch and guar gum during frying. The starch granules swell, release the amylose contents, and provide a film (together with guar gum) that inhibits or reduces moisture loss and oil diffusion from the fried product (Arshad et al., 2019). In addition, Mudau et al. (2021) stated that the increase in moisture content may be due to high fiber content. Guar gum has been observed to be high in fiber. SGG and SX samples had high moisture content and were not cracked with a dry crumb compared to SC. The WC had 37.33% compared to the SC, which had 33.12%, thus having a significant difference (*p* < .05). Bhagwan et al. (2011) and Liu et al. (2020) observed that hydrocolloid incorporation into a food can affect moisture content migration. A further observation was made that with an increase in hydrocolloid level, there was a notable increase in the moisture content. Hydrocolloids act as obstacles to the migration of moisture from a fried product. The moisture barrier power might reduce the weight loss of the glazed food product (Bourlieu et al., 2009; Sason & Nussinovitch, 2021).

The SC had the lowest moisture content (Table 3). This may be linked to the rapid moisture loss during the frying process. Comparing the SX and SGG values, the SX values decreased significantly with an increase in xanthan gum. In contrast, SGG values increased significantly with an increase in guar gum. Factors such as moisture content, movement, shifting of water in a product, and gluten‐starch interactions affect the hardness of baked and fried foods (Eriksson et al., 2014). The high moisture content (Table 3) in the SX and SGG samples meant that the hydrocolloids interacted well with the sorghum flour molecules, thus holding water in the product, and redistribution of water occurred during frying.

**TABLE 3:** Proximate composition (%) of sorghum *magwinya* (fried dough).

Samples	Moisture	Oil	Fiber	Ash
WC	37.33^cd^ ± 2.31	12.06^c^ ± 0.46	1.77^a^ ± 0.16	1.38^a^ ± 0.04
SC	33.12^a^ ± 1.52	17.26^d^ ± 1.05	2.89^d^ ± 0.28	1.70^b^ ± 0.05
SX0.5	34.67^abc^ ± 1.15	12.01^c^ ± 0.61	2.42^bc^ ± 0.25	2.01^c^ ± 0.06
SX1.0	36.67^bcd^ ± 1.54	10.25^b^ ± 0.63	2.55^bcd^ ± 0.15	2.07^c^ ± 0.08
SX1.5	37.33^cd^ ± 1.15	10.12^b^ ± 0.44	2.79^cd^ ± 0.12	2.07^c^ ± 0.03
SGG1.5	34.00^ab^ ± 2.00	9.78^ab^ ± 0.81	3.64^e^ ± 0.07	2.59^e^ ± 0.17
SGG2.0	36.42^bcd^ ± 1.42	9.61^ab^ ± 0.94	2.27^b^ ± 0.23	2.23^cd^ ± 0.40
SGG2.5	39.06^d^ ± 1.83	8.48^a^ ± 0.13	2.59^bcd^ ± 0.35	2.40^de^ ± 0.16

*Note*: Values are the mean of three replications with the standard deviations. Superscripts in the same column with different alphabets significantly differ (*p* < .05). WC and SC are wheat and sorghum control. SX (0.5, 1.0, and 1.5) and SGG (1.5, 2.0, and 2.5) represent xanthan (SX) and guar gum (SGG) variations in the sorghum samples.

### 4.6 The oil content of *magwinya*

One of the mechanisms of oil uptake in fried products is capillary action. When the sorghum *magwinya* dough is immersed in hot oil, the pores and channels allow the oil to penetrate the dough through capillary action (Yazdanseta et al., 2015). Effects of the levels of the two hydrocolloids on the fat content of *magwinya* were studied, and the results obtained were recorded. Hydrocolloids change the volume of the water held, and as a result, they affect oil absorption (Varela et al., 2016). Bhagwan et al. (2011) observed that the oil content of samosas notably reduced with an increase in the level of hydrocolloids, irrespective of the kind of hydrocolloid used. SC had the highest oil content (17.26%). When sorghum starch and guar gum were added to the dough, an instant gel formation might have occurred upon contact, potentially inhibiting the uptake of oil (Arshad et al., 2019). Therefore, it is probable that bonding and cross‐linking between hydrogen or disulfide bonds of the starch molecules and the hydrocolloids took place. Varela and Fiszman (2011) stated that hydrocolloids may contribute to the reduction of porosity of the fried dough. Thus, a less porous structure means fewer openings for oil uptake. In this study, SGG dough had likely formed a gel that obstructed and reduced the oil uptake. The oil uptake reduction in the SGG samples might be linked to their viscosity properties effect (Sahin et al., 2005). Table 2 shows that the oil content of *magwinya* reduced as the level of hydrocolloid increased. The oil content of *magwinya* decreased significantly in XG1.5 (10.25%) and SGG2.5 (8.48%) samples. The reduction of the oil uptake in the SGG samples might have been linked to their viscosity‐building effect (Sahin et al., 2005). Between the hydrocolloids used, guar gum with a 2.5 g concentration level was statistically effective in reducing oil content in *magwinya*. This might have been due to the formation of films within the *magwinya*, which could have decreased the tendency of *magwinya* to absorb the oil and lose moisture (Annapure et al., 1999). Hydrocolloids are water‐soluble polysaccharides with various chemical structures. This might help hydrocolloids create a barrier to oil absorption while retaining the natural moisture of foods (Mir et al., 2016). SC (33.12%) had the lowest moisture content but the highest oil content (17.26%). A similar observation was reported by Arshad et al. (2019), where doughnut samples with lower moisture content were seen to have higher fat content after frying. SGG *magwinya*, with the lowest fat content, meets the request for low‐fat foods by health‐conscious consumers.

### 4.7 Fiber content of *magwinya*

Sorghum components might influence metabolic disease by slowly delivering resistant and digestible starches, dietary fiber, policosanols, polyphenols, unsaturated fatty acids, and high antioxidant volume (Stefoska‐Needham et al., 2015). High‐fiber‐content foods might reduce the risks of celiac disease and colon cancer by boosting metabolism (Onipe et al., 2015). Incorporating the hydrocolloids increased the total dietary fiber of *magwinya* from 1.77% (WC) to 3.64% (SGG). The sorghum *magwinya* samples also had high fiber content compared to wheat because sorghum is rich in dietary fiber, increasing the total dietary fiber (TDF) content of the samples (Kaplan et al., 2020). The fiber content of SC (2.89%) was notably higher than WC (1.77%). Guar gum is extracted from seed and endosperm of a cluster bean, thus making it higher in fiber than xanthan gum, which is produced from microorganisms (Ebrahim et al., 2021). As anticipated, fiber content increased significantly (*p* < .05) with guar gum incorporation in *magwinya*. SGG1.5 (3.64%) had the highest significant value among the other samples. This study shows that guar gum was an excellent hydrocolloid due to its positive impact on *magwinya* fiber content.

### 4.8 Ash content of *magwinya*

The ash content of *magwinya* with hydrocolloids was in the range of 2.01%–2.40%, and they showed notable differences within the other samples and from the ash value of WC (1.38%). There was no significant difference between the hydrocolloid levels in SX, but a notable difference was seen between the SX and SGG. The estimate of ash content is equivalent to the total mineral content in a food sample (AACC, 2000). The sorghum kernel is concentrated with essential nutrients, and it is a gluten‐free crop containing many vitamins and minerals, making it an excellent dietary choice (Renzetti et al., 2008). Compared to the WC (1.38%), SC (1.70%) had a high ash content value, and there was a significant increase in ash content with the addition of the hydrocolloids in sorghum. This shows how nutritious and nutrient‐packed sorghum *magwinya* is compared to wheat *magwinya*. The ash content corresponds to the mineral content.

## 5 CONCLUSION

The hydrocolloids showed remarkable binding results and the capability to form a well‐shaped, nutritious sorghum *magwinya*. *Magwinya* produced with the incorporation of guar gum showed good results in oil uptake, ash content, fiber, and hardness. *Magwinya*, with low fat, high fiber and nutrient content, can meet the demand for gluten‐free, low‐fat food for health‐conscious consumers on a gluten‐free diet. As guar gum is derived from the seed endosperm of the guar plant, this explains the high fiber and ash content seen in this study. Considering all the results obtained in this study, sorghum *magwinya* incorporated with guar gum can be suggested to be used, thus substituting for the normal wheat *magwinya*. The main emphasis of this study was placed on examining the firmness of *magwinya* due to its significant association with consumer satisfaction or preference. Therefore, it is recommended to analyze the gumminess, cohesiveness, springiness, and chewiness textural parameters alongside hardness, which would provide a broader understanding of the texture profile of *magwinya*, and an understanding of how these different parameters interact to contribute to the overall consuming feel and quality of the product. Sensory evaluation on sorghum *magwinya* is also recommended to see consumer acceptance and to provide a direct link between the technical results and potential market acceptance. Other hydrocolloids like pectin, carboxymethylcellulose (CMC), and hydroxypropyl methylcellulose (HPMC) are also used in food processing. It is, therefore, necessary to investigate the incorporation of other hydrocolloids in sorghum *magwinya* production. Comparative analysis with other non‐wheat flours like finger millet, rice, and chickpea flours is recommended. It might highlight the diverse impacts of different gluten‐free flours on the physicochemical properties and overall quality of fried products like *magwinya*.

## AUTHOR CONTRIBUTIONS

**Oluwatoyin O. Onipe:** Methodology (lead); supervision (supporting); writing – review and editing (lead). **Afam I. O. Jideani:** Conceptualization (lead); funding acquisition (lead); project administration (lead); resources (lead); supervision (lead); writing – review and editing (lead).

## CONFLICT OF INTEREST STATEMENT

The authors declare that there is no conflict of interest.

## Data Availability

The data supporting the conclusions of this article are presented therein.
